# Antimicrobial drug resistance against *Escherichia coli* and its harmful effect on animal health

**DOI:** 10.1002/vms3.825

**Published:** 2022-05-24

**Authors:** Safia Arbab, Hanif Ullah, Weiwei Wang, Jiyu Zhang

**Affiliations:** ^1^ Key Laboratory of Veterinary Pharmaceutical Development Ministry of Agriculture Lanzhou China; ^2^ Key Laboratory of New Animal Drug Project of Gansu Province Lanzhou China; ^3^ Lanzhou Institute of Husbandry and Pharmaceutical Sciences Chinese Academy of Agricultural Sciences Lanzhou China; ^4^ West China School of Nursing Sichuan University Chengdu China

**Keywords:** animal health, antimicrobial resistance, *Escherichia coli*, gram‐negative bacteria, harmful effect

## Abstract

Multidrug resistance among pathogenic bacteria is imperilling the worth of antibiotic infection, which has become an emerging problem, which previously transformed the veterinary sciences. Since its discovery, many antibiotics have been effective in treating bacterial infections in animals. *Escherichia coli*, a bacterium, is one of the reservoirs of antibiotic resistance genes in a community. The current use of antibiotics and demographic factors usually increase multidrug resistance. Genetically, the continuous adoption of environmental changes by *E. coli* allows it to acquire many multidrug resistance. During the host's life, antimicrobial resistance rarely poses a threat to the *E. coli* strain and pressure, similar to that of a flexible animal lower intestine. In this review, we describe the *E. coli* antibiotic drug–resistance mechanism driving transmission, the causes of transmission and the harmful effects on animal health.

## 1 INTRODUCTION

Antibiotic resistance refers to a host's drug‐susceptible response to a microbial infection and it has become an emerging problem. Antibiotic resistance in microbes is a major health concern all over the world, particularly in third world countries. Due to poor waste and water management, humans are in continuous contact with microbes and disease‐causing bacteria (Afzal, 2017). The excessive use of antibiotics introduces a selective pressure that is becoming responsible for resistance or even multiresistance characteristics in some bacterial populations (Chen & Jiang, 2014). The treatment of infections caused by antimicrobial‐resistant pathogens challenges health care systems worldwide, significantly increasing costs and placing a huge burden on resources (French, 2005). Thus, the emergence of antimicrobial resistance (AMR) has become a grave concern due to its impact on patients, physicians, health care systems and pharmaceutical producers (McGowan Jr, 2001).

*Escherichia coli* is the most prevalent facultative anaerobic species in the gastrointestinal tract of humans and animals, and is usually a harmless microbe, but it is also a medically important bacterium causing a number of significant illnesses (Akbar & Anal, 2011; Friedman et al., 2002). *E. coli* are normally found in the intestinal tract of humans and warm‐blooded animals, but some strains have acquired pathogenic or toxigenic virulence factors that make them virulent to humans and animals (Malik & Memona, 2010). A high prevalence of *E. coli* poses a potential risk to animals and human health (Arbab et al., 2021b, 2021c).

Antibiotics are often used for the therapy of infected humans and animals as well as for prophylaxis and growth promotion of food‐producing animals. Many findings suggest that inadequate selection and abuse of antimicrobials may lead to resistance in various bacteria and make the treatment of bacterial infections more difficult (Kolár et al., 2001). Treatment for *E. coli* infection has become increasingly complicated because of the emergence of resistance to most first‐line antimicrobial agents (McKeon et al., 1995).

The accumulating effect of traditional antimicrobials was completed by the continuous discovery and introduction of new therapeutic drugs, which drives bacteria to be trained to constantly change by selecting appropriate AMR phenotypes and genotypes. Once armed with the required set of AMR genes, bacterial strains may have the advantage of surviving and spreading both in animal and human populations, since with few exceptions, the same antimicrobial classes are used to treat infections in animals and humans (Courvalin, 2006).

Although antimicrobial classes are common in veterinary medicine, their importance may vary according to the species and application. The majority of antibacterial compounds are generally used to treat a wide range of animals and infections, but there are drugs with applications restricted to certain groups of species (e.g., Difloxacin to avian infections). On the other hand, some antimicrobial classes, such as cephalosporins (first to fourth generation), are represented by a large number of compounds for treating serious infections in humans, while only a few of them have the veterinary application of the World Organization for Animal Health (OIE) (WHO, 2007). Here, we provide a brief overview of the history of antibiotics, the *E. coli* antibiotic drug‐resistance mechanism driving transmission, the causes of transmission and its harmful effects on animal health.

## 2 A BRIEF HISTORY OF ANTIBIOTICS

The use of antibiotic‐producing microbes to prevent disease stretches back to millennia, with traditional poultices of mouldy bread being used to treat open wounds in Serbia, China and Greece more than 2000 years ago. Both are broad‐spectrum antibiotics as they work against gram‐negative bacteria. (Brunel, 1951). The production of diffusible and heat‐stable compounds by bacteria was reported by the turn of the 20th century, and their utility in combatting infectious diseases has been explored. Dorothy Hodgkin solved the β‐lactam structure of penicillin in 1945 (Hodgkin, 1949). Resolving the famous debate between Robert Robinson, who favoured a thiazolidine‐oxazolone structure, and several other notable chemists, including Chain, Abrahams and Woodward, who believed it to be a β‐lactam (Curtis & Jones, 2007). This was an important breakthrough because it enabled the development of semisynthetic derivatives to bypass penicillin resistance. Arguably, the first clinical use of an antibiotic was reported in the 1890s, where Emmerich and Low used an extract of *Pseudomonas aeruginosa* (then known as *Bacillus pyocyaneus*) to treat hundreds of patients, and this extract, called *pyocyanase*, was used until the 1910s (*Emmerich & Löw*, *1899*). *Pyocyanase* was active towards multiple pathogens and incorrectly believed to be an enzyme. Instead, the active component of *pyocyanase* was likely to be a mixture of pyocyanin, a quorum‐sensing phenazine and 2‐alkyl‐4‐hydroxyquinolones (Hays et al., 1945).

## 3 MECHANISMS OF ANTIBIOTIC RESISTANCE

**FIGURE 1:**
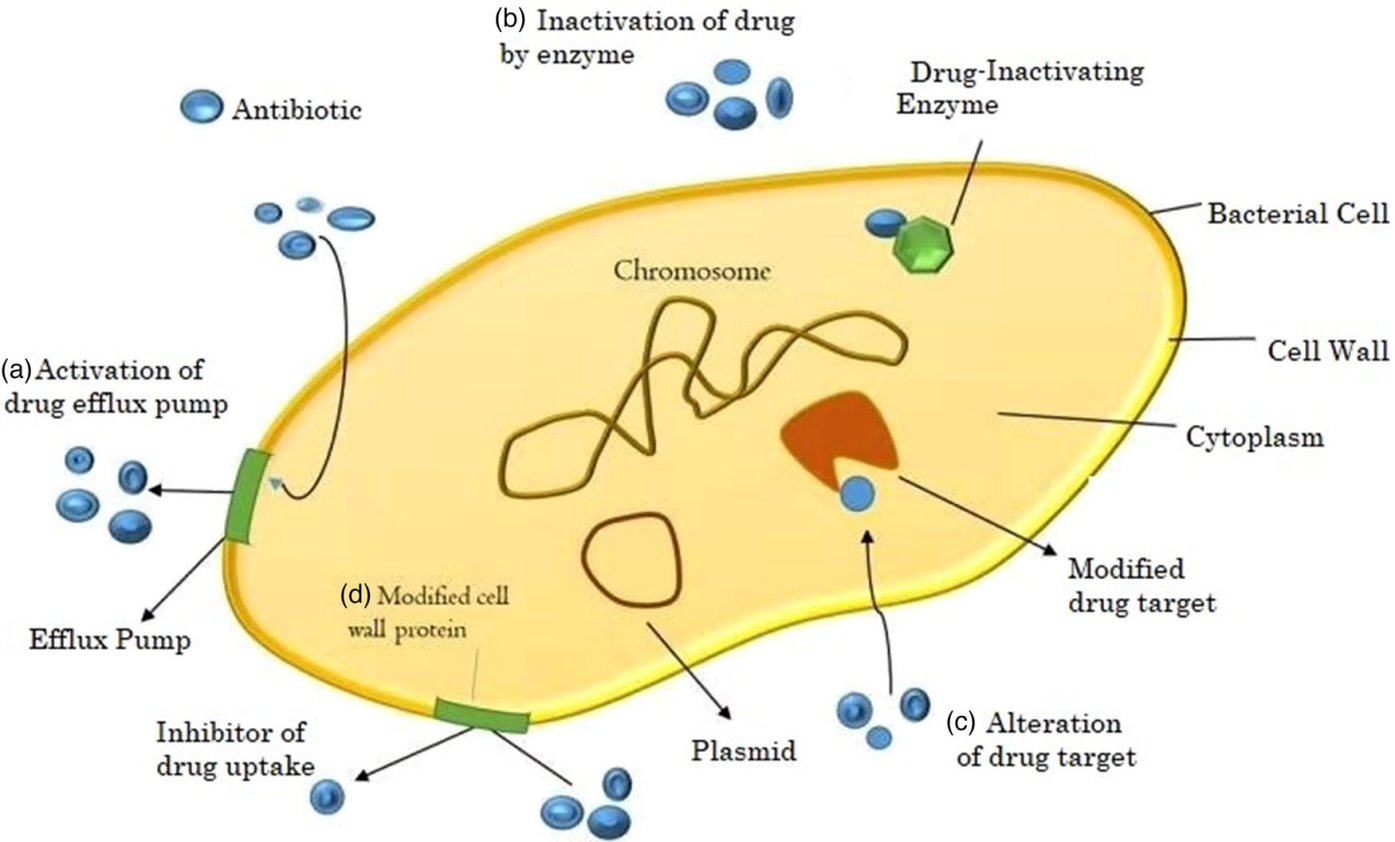
Mechanisms of resistance in bacteria. Showing the bacterial cell representative figure of the mechanisms that confer resistance to drugs; (a) efflux pump in which drug vigorously pumps out of the cell; (b) enzymatic degradation in which the enzyme immobilises the drug; (c) modification of the target molecules—when the drug is no longer connected to its target molecules and (d) they are transformed into the membrane permeability appropriate for the porous boundaries attached to the membrane or for the change of their forms modified by (Bbosa et al., 2014)

There are four general AMR mechanisms that bacteria use. These are limiting uptake of the drug, modifying the target of the drug, inactivating the drug and active efflux of the drug. These mechanisms may be located on the bacterial chromosome and occur naturally in all members of a species (intrinsic) or come from other bacteria, usually via a plasmid (acquired) route. Intrinsic resistance genes may be expressed constitutively (usually at a low level) or be induced by the presence of antimicrobial drugs. Gram‐negative bacteria widely use all four of these mechanisms and are capable of horizontal transfer of resistance elements (Mehrad et al., 2015; Reygaert, 2016). Surprisingly, this is still true for many other pathogenic bacteria, including *Enterobacteriaceae*, which have become resistant not only to the original penicillin but also to semisynthetic penicillins, cephalosporins and newer carbapenems (Kumarasamy et al., 2010). Figure 1 describes that the bacterial cell can use the efflux pump. The gram‐negative bacterial cell can use the outlet characterised pump A or change the membrane permeability D to obtain the maximum value quantity of drug near the cell, and the antibiotic can be inactivated for all the enzymatic action B in or maximise the ease with which it binds to its target site C. It is not atypical for a particular group to demonstrate more than one of these mechanisms (Meletis & Bagkeri, 2013).

## 4 ANTIBIOTIC RESISTANCE SPREAD AND DIVERS TRANSMISSION

**FIGURE 2:**
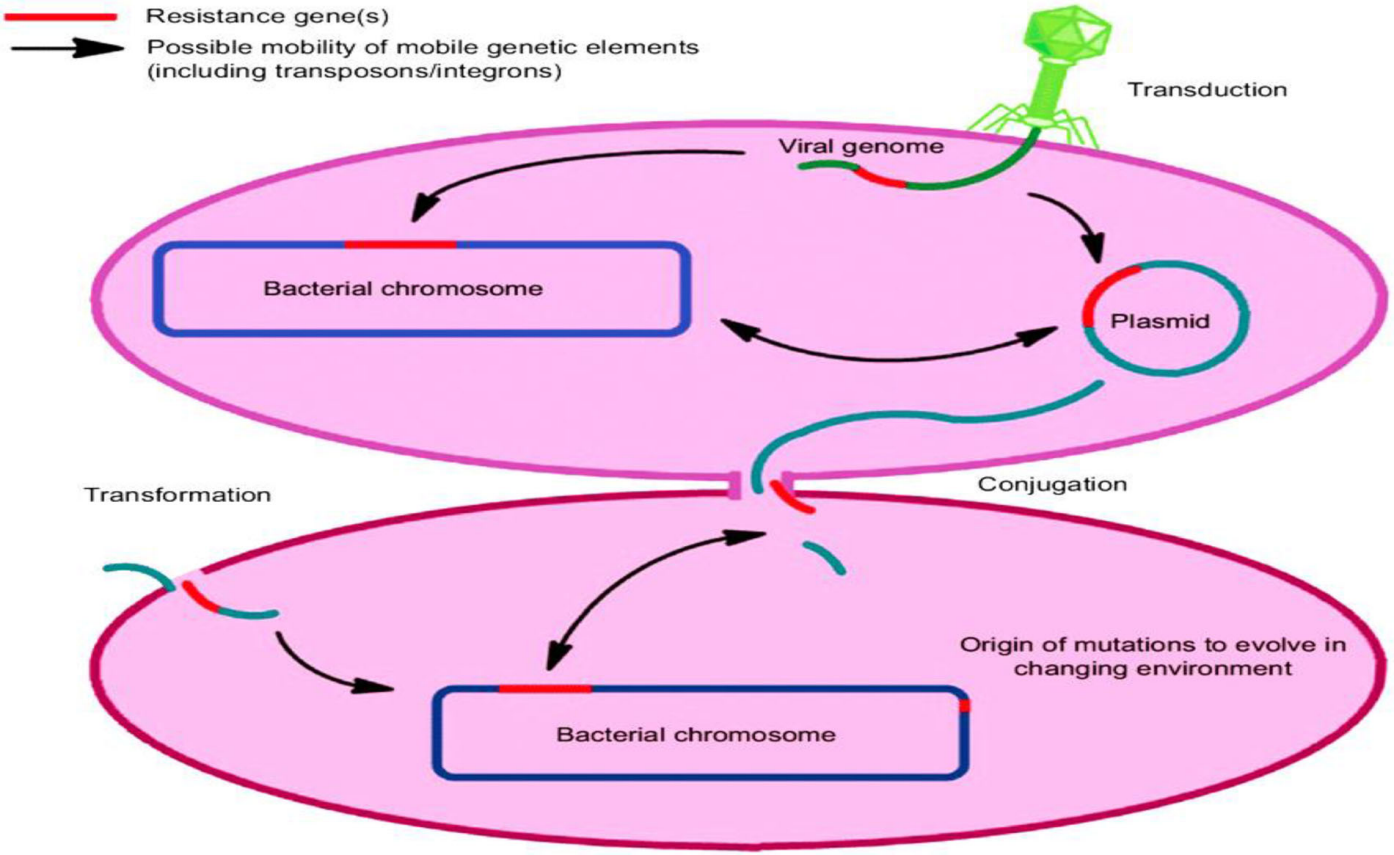
Drivers of antibiotic resistance transmission (Aslam et al., 2018)

At the moment, understanding several factors that determine antibiotic resistance is the key to addressing this global concern. The emergence of resistance in microbes is a natural process (Figure 2); until now, the selection of antibiotic resistance has been carried out using different antibiotics in health systems, in the environment and in agriculture/livestock. Additional essential factors that are powerful drivers of antibiotic resistance include health care environments, infection control standards, water hygiene systems, drug quality, diagnosis and therapy and travel or migration quarantine. In addition to mutations in several genes residing on the chromosome of the microorganism, the exchange of genetic material between organisms plays a vital role in the distribution of antibiotic resistance. Plasmid transmission is an essential phenomenon that can transfer genes from antibiotic resistance to “guestMobile.” Antibiotics can influence this process by inducing the transmission of resistance elements; these antimicrobials can also exert selective pressure on the occurrence of resistance (Munita & Arias, 2016). The rate and degree of increase in resistance varies widely with different pathogen‐drug combinations and geographical locations (Morley et al., 2020). Many studies have focused on developing effective treatments for combatting infections caused by resistant isolates, such as by modifying dosage or through the use of another class of antibiotics (Ventola, 2015b). The emergence of some rare isolates of gram‐positive and gram‐negative bacteria that exhibit resistance to a wide range of antibiotics, such as vancomycin, in resistant *Enterococci* and multiresistant gram‐negative bacteria is a cause of concern (Cantas et al., 2013). Understanding how resistance genes and the bacteria that harbour them spread is critical to controlling this ongoing global issue. Bacterial resistance can be intrinsic or acquired. Intrinsic resistance is mediated chromosomally, while acquired resistance may be due to one or more mutations or the acquisition of plasmids and other transposable DNA elements (Chinedum, 2005). Intrinsic resistance is known and predictable because it is based on the inherent properties of the species (Chinedum, 2005). The worldwide spread of gene resistance in pathogens depends on the transmission of resistance genes within microbial populations. The ability of an organism (carrier genes or low resistance genes) to survive and reproduce in a particular environment is often called body strength. An organism's attitude is formed called by the additional elements of the chromosomal liquid it carries and the available substrates (Smith & Bidochka, 1998). The first important step in predicting the importance of AMR for food safety, animal health and health is to describe the association between the different resistances. The prevalent spread of the same multidrug resistance (MDR) phenotypes that mediate the “oldest” AMR has also been reported in consecutive European surveys conducted to check antimicrobial susceptibility in *E. coli* isolates from healthy animals (Bywater et al., 2004; de Jong et al., 2011). A similar study reported that the association between ampicillin, doxycycline and tetracycline‐sulfamethoxazole/trimethoprim is predominant in poultry and pig production in China (Jiang et al., 2011).

## 5 RESISTANCE OF ANTIBIOTIC CAUSES

Currently, the multifaceted aetiology of antibiotic resistance has many factors. These include inadequate regulations and inaccuracies in use, lack of awareness of best practices that address the misuse or inappropriate use of antibiotics, use of antibiotics as a promoter of poultry and livestock growth instead of controlling the infection and online marketing, which made the unlimited availability of low‐quality drugs very accessible. Mainly, the overuse of antibiotics is the main cause of the evolution of resistance. Antibiotics kill sensitive bacteria but allow resistant pathogens to remain reproducing and thrive through natural selection. Although the overuse of antibiotics is discouraged, there is still an excessive prescription worldwide. Several studies have revealed that indications of treatment, choice of agent and duration of antibiotic therapy are inadequate in 30%‐50% of cases (Read & Woods, 2014; Ventola, 2015a). Globally, antibiotics are used as growth promoters in cattle. According to one estimate, approximately 80% of antibiotics are sold in the United States for use as growth supplements only and to control infection in animals. In another study, a global map of 228 countries was drawn representing the consumption of antibiotics in cattle; total antibiotic use was estimated to have been 63,151 tons in 2010 (Van Boeckel et al. 2015). Van Boeckel et al (2015) also predicted a 67% increase in antibiotic use by 2030, which would roughly double in Brazil, Russia, India, China and South Africa (developing and highly populated countries of the world) (Van Boeckel et al., 2015).

## 6 ANTIBIOTIC RESISTANCE IN *E. COLI*

*E. coli* are a widespread bacterial species that comprise a broad variety of strains and can be highly pathogenic (Tareen et al., 2022). Drug‐resistant *E. coli* infections extend the length of stay in hospitals, which poses economic pressure directly and indirectly over the population and health care systems (MacKinnon et al., 2020). Antibiotic resistance is a phenomenon in which some subpopulations of bacteria resist the presence of one or more antibiotics, and pathogens that are resistant to multiple antibiotics are considered multidrug‐resistant (MDR) or superbugs (Chowdhary et al., 2014). The evolution of resistant bacterial strains is a natural phenomenon that occurs when microorganisms replicate themselves erroneously or when resistance traits are exchanged between strains through horizontal gene transfer mechanisms. Bacterial resistance to antibiotics is increasingly becoming a concern to animal health. Currently used antibiotic agents fail to end many bacterial infections due to super‐resistant strains (Arbab et al., 2021d). The use and misuse of antimicrobial drugs accelerate the emergence of drug‐resistant strains. Poor infection control practices, inadequate sanitary conditions and inappropriate food handling encourage the further spread of AMR (WHO, 2004). Moreover, the scenario of AMR is not restricted to human pathogens but is also common in veterinary pathogens. It has been reported that extended‐spectrum β‐lactamase‐ and metallo‐β‐lactamase‐producing strains are common in animals and present in their environment (Singh et al., 2012). It was found that in hospitals, plasmid‐directed mutations are very high, leading to antibiotic resistance in *E. coli*. Previously, antibiotic resistance of *E. coli* was tested and found to be nearly 70% against streptomycin sulfsoxazole‐tetracycline. It was also shown that ampicillin, kanamycin, sulfsoxazole, streptomycin, tetracycline and ticarcillin have a decline in susceptibility (Tadesse et al., 2012).

Often, antibiotics are used to treat humans, as well as food‐producing animals. Numerous results recommend that improper use of antimicrobials can lead to the resistance of many bacteria and make it challenging to treat harmful bacterial infections (Arbab et al., 2021a). *E. coli* AMR has been reported worldwide. *E. coli* infection was completed with the prescription of antibiotic‐resistance agents (Momtaz et al., 2012). Cephalosporin among enterobacterial members increased mainly due to the spread of broad‐spectrum antibiotics (Potron et al., 2015).

The creation of mechanisms of resistance to β‐lactamases frequently frequent bacteria against β‐lactam drugs (Arbab et al., 2021c). Initially, *E. coli* characterised chromosomally encoded β‐lactamases and ampC from the gene (called ampicillin resistance). Increasing resistance to antimicrobial drugs is among the world's most critical concerns. For many developing countries, the possibility of antibiotics is a problem with AMR, with most pathogenic bacteria and practically all opportunistic infections caused by bacteria. During the accumulation of the disease, there is a higher mortality rate than antimicrobial drugs (Haenni et al., 2014).

*E. coli* characterisation determinates are common resistance type animals based on some type of *E*. *coli* gene are, blaTEM ampicillin, broad‐spectrum drug type tetracycline and streptomycin and trimethoprim type, in addition to these primary listed genes, more than a few additional genes have been identified that encode the same resistance phenotype: blaOXA‐1 ampicillin, sul2 sulfamethoxazole and trimethoprim (Bonnet et al., 2009; Guerra et al., 2003; Szmolka et al., 2012). Several animal studies have shown several examples of drug resistance to bacterial infection, one of the first descriptions of strains in wild animals (Costa et al., 2006). CTX‐M, TEM and SHV indicate the occurrence of extended‐spectrum β‐lactamase classes (Jacoby & Munoz‐Price, 2005). Several studies focusing on wild boar have reported one and level two integrons with their natural resistance genetic (Mokracka et al., 2012), a new range of extended‐spectrum β‐lactamases and determinants of nalidixic acid resistance and ciprofloxacin in Portugal (Poeta et al., 2009). Similar results from a wide range of wild mammals have been reported in the Czech Republic/Slovakia (Literak et al., 2010).

## 7 *E. COLI* AND ITS HARMFUL EFFECT OF ANIMAL HEALTH

*E. coli* are the most crucial part of the bacteria that are generally present in healthy intestines. Different strains of *E. coli* are not harmless, except that some strains of *E. coli* cause harmful effects in humans, birds and animals (Kaper et al., 2004). Cattle and ruminant's most crucial reservoir *E. coli* O157: H7 (Ferens & Hovde, 2011; Munns et al., 2015), some related studies have approximately 75% 0157:H7: of the human epidemic *E. coli* bovine food‐producing animals (Callaway et al., 2009). Additional *E. coli* causes of transmission include sheep (Gencay, 2014), goats (Pao et al., 2005; Swift et al., 2017), deer (Renter et al., 2001) and some species of bird (Wetzel & LeJeune, 2006). An investigation of 390 occurrences of *E. coli was* reported in 2003 in the United States, and 65% found that 2012 involved food transmission and transmission with animals, person‐to‐person (Heiman et al., 2015). Bovine animals frequently continuously spread infection in the environment (Gansheroff & O'Brien, 2000; Sperandio & Nguyen, 2012). Surrounded by cattle, shedding occurs intermittently (Kulow et al., 2012; Sharma et al., 2012). Most *E. coli* isolates reside harmlessly in the gastrointestinal tract of humans and animals as part of their normal microflora and benefit the hosts by producing key nutrients, such as vitamin K (Suvarna et al., 1998). Some isolates of *E. coli* are able to colonise sites outside the intestine and cause extraintestinal disease (Sharma et al., 2007). For example, *E. coli* is also recognised as a major cause of urinary tract infections (UTIs) that can lead to the development of acute cystitis and pyelonephritis (DeCory et al., 2005).

## 8 CONCLUSIONS

Antimicrobial drug resistance in *E. coli* is an issue of the utmost importance since it occurs in both humans and animals. In animals, MDR in *E. coli* may lead to difficult‐to‐treat infections, but even more importantly, it constitutes a major and shared reservoir of resistance determinants to most families of antimicrobial agents. The antimicrobial appearance of bacterial pathogenic agents has become a major issue for animal health. Antimicrobial drug resistance also repeatedly challenges the adaptive intestinal tract of the *E. coli* strain during the life of the host. At national, regional and global levels, monitoring, bio vigilance and response and prevention strategies for AMR and MDR pathogens can help control animal health risks.

## CONFLICTS OF INTEREST

The authors declare that there are no conflicts of interest.

## FUNDING INFORMATION

The work has been supported by the earmarked fund for the National Natural Science Foundation of China (No: 31872520) and China Agricultural Research System (CARS‐37). The authors would like to thank Professor Jiyu Zhang, PhD, Lanzhou Institute of Husbandry and Pharmaceutical Sciences, Chinese Academy of Agriculture Sciences, for providing feedback on this study.

## AUTHOR CONTRIBUTIONS

Safia Arbab: Conceptualisation, formal analysis, manuscript writing. Hanif Ullah: Writing—review and editing. Jiyu Zhang: Funding acquisition, investigation, project administration, supervision. Weiwei Wang: Review and editing.

## ETHICAL STATEMENT

This review was conducted according to the guidelines under approved by the Animal Administration and Ethics Committee of Lanzhou Institute of Husbandry and Pharmaceutical Sciences, Chinese Academy of Agricultural Sciences. The certificate number was SCXK (Gan) 2019–001.

### PEER REVIEW

The peer review history for this article is available at https://publons.com/publon/10.1002/vms3.825.

## Data Availability

All the relevant data are available in the manuscript.
